# UCHL1-PKM2 axis dysregulation is associated with promoted proliferation and invasiveness of urothelial bladder cancer cells

**DOI:** 10.18632/aging.205097

**Published:** 2023-10-09

**Authors:** Yuhui Zheng, Dongliang Shi, Linlin Chen, Yinghong Yang, Meihong Yao

**Affiliations:** 1 Department of Pathology, Fujian Medical University Union Hospital, Fuzhou 350001, Fujian, China

**Keywords:** UCHL1, PKM2, bladder cancer, urothelial bladder carcinoma

## Abstract

Background: Bladder cancer is one of the most common type of cancers globally, and the majority of cases belong to urothelial bladder carcinoma (UBC) type. Current researches have demonstrated that multiple genomic abnormalities are related to the sensitivity of cisplatin-based chemotherapy in bladder cancer patients. Previous findings have indicated a controversial role of Ubiquitin Carboxy-Terminal Hydrolase L1 (UCHL1) in malignancy, so we aimed to further explore the role of UCHL1 in UBC.

Methods: UBC cell lines and The Cancer Genome Atlas (TCGA) *in-silico* datasets were utilized to investigate UCHL1 expression pattern and functional as well as prognostic impacts in UBC cancer cell line models and patients. UCHL1 overexpression and silencing vectors and subsequent immunoprecipitation/ubiquitination experiments in combination of cellular functional assays were conducted to explore UCHL1-PKM2 interaction axis and its significance in UBC malignancy.

Results: UCHL1 was significantly up-regulated in UBC cancer cells and UCHL1 high-expression was associated with higher pathology/clinical grade and significantly inferior overall prognosis of UBC patients. UCHL1 interacted with PKM2 and enhanced PKM2 protein level through inhibition of PKM2 protein degradation via ubiquitination process. UCHL1-PKM2 interaction significantly promoted UBC cellular proliferation, metastasis and invasion activities.

Conclusion: UCHL1-PKM2 interaction played an interesting role in UBC tumor cell proliferation, migration and metastasis. Our study suggests PKM2-targeted treatment might have a potential value in metastatic malignancy therapy development in the future.

## INTRODUCTION

Bladder cancer is one of the most common type of cancers globally, with average 430,000 newly diagnosed cases annually [1]. Among them, more than 80% of bladder cancer cases are pathologically diagnosed as the urothelial bladder carcinoma (UBC) type [2], and others tumor types include squamous-cell carcinoma, adenocarcinoma, etc. Based on the tumor invasion pattern, bladder cancer can be also classified into muscle-invasive bladder cancer (MIBC) and non-muscle-invasive bladder cancer (NMIBC) [3]. And the prognosis of MIBC patients are much worse compared with NMIBC patients, due to the high risk of cancer metastasis. For MIBC patients, the most utilized therapy contains radical cystectomy combined with chemoradiotherapy [4–6], and transurethral resection followed by intravesical chemotherapy is often selected as a standard-of-care (SoC) for NMIBC patients [7–9]. Platinum-based neoadjuvant chemotherapy has also been gradually considered as a SoC due to the improvement of patients’ overall survival [10–12].

Over the past decades, researches have reported multiple genomic characteristics which are related to the sensitivity of cisplatin-based chemotherapy in bladder cancer patients. For example, recurrent somatic mutations of ERCC2, ATM, RB1 and FANCC have all been considered to have a predictive value in chemotherapy response evaluation for MIBC patients [13, 14]. Besides, other molecular studies also indicated that bladder cancer cells can be classified by transcriptional signatures which are reflective of tumor cell differentiation degree. For instance, KRT5/KRT14 fingerprints are associated with basal-type tumors while FOXA1/GATA3 fingerprints are associated with luminal-type tumors [15, 16]. Based on the previous achievements, it will be of significant value to further delineate the heterogeneity of bladder cancer in order to enhance bladder cancer treatment response and patients’ survival.

Ubiquitin Carboxy-Terminal Hydrolase L1 (UCHL1) belongs to the UCH family of deubiquitinases (DUBs) which are responsible for ubiquitin isopeptide bond elimination from target proteins. Current understanding of UCHL1’s role is in debate, and previous study suggests that UCHL1 regulates estrogen receptor (ER) and transforming growth factor beta (TGF-β) pathways, and UCHL1 dysregulation is associated with development of breast cancer aggressiveness [17]. Therefore, in this study, we aim to further explore the impact of UCHL1 expression on UBC malignancy.

## MATERIALS AND METHODS

### UBC clinical samples

In general, 36 pairs of matched-samples of UBC tumor and adjacent normal tissues were collected respectively at the clinical center. The informed consent from patients have been obtained. UBC tumor RNA-seq datasets and corresponding patients’ clinical data were retrieved in platform TCGA. The study was approved by the Ethics Committee of the Fujian Medical University Union Hospital and informed consent from each patient was required for study enrollment. Pathological diagnosis for all patients were confirmed by two independent pathologists based on sample HE staining and immunohistochemistry assay results. The inclusion criteria: patients pathologically diagnosed as UBC.

### Cell culture, vectors and transfection

UBC cancer cell lines UM-UC-3, SW780, T24, J82, BIU-87, 5637 (ATCC, Manassas, VA, USA) were acquired from the Fujian Medical University Union Hospital. All types of tumor cell lines were cultured utilizing RPMI-1640 medium (Gibco, Langley, OK, USA) combined with 10% fetal bovine serum (Gibco, Langley, OK, USA). All cells were cultured at 37°C with 5% CO_2_ incubator environment. Vector transfection was conducted using Lipofectamine 3000 Reagent (Invitrogen, Waltham, MA, USA), and transient transfection was constructed.

### RNA isolation and real time PCR

Total RNA was firstly extracted by TRIzol reagent (#R0016, Beyotime, Beijing, China) and was subsequently used for RT utilizing primers and the 5 × All-In-One kit (Abcam, Cambridge, UK). Afterwards, RNAs were quantified SYBR Green Master Mix. GAPDH was utilized as an endogenous control, all primers used in this study are listed as follows.

**Table:** 

UCHL1:	
Forward	5′-GAAGCAGACCATCGGAAACTCC-3′,
Reverse	5′-GGACAGCTTCTCCGTTTCAGAC-3′;
PKM2:	
Forward	5′-ATGGCTGACACATTCCTGGAGC-3′,
Reverse	5′-CCTTCAACGTCTCCACTGATCG-3′;
E-Cadherin:	
Forward	5′-GCCTCCTGAAAAGAGAGTGGAAG-3′,
Reverse	5′-TGGCAGTGTCTCTCCAAATCCG-3′;
N-Cadherin:	
Forward	5′-CCTCCAGAGTTTACTGCCATGAC-3′,
Reverse	5′-GTAGGATCTCCGCCACTGATTC-3′;
Vimentin:	
Forward	5′-AGGCAAAGCAGGAGTCCACTGA-3′,
Reverse	5′-ATCTGGCGTTCCAGGGACTCAT-3′;
GAPDH:	
Forward	5′-GTCCATGCCATCACTGCCAC-3′,
Reverse	5′-AAGGCTGTGGGCAAGGTCAT-3′.

### Colony formation assay

For colony formation assay, UBC cells from each treatment group were placed into 6-well plates with density of 1 × 10^3^ cells per well. Then tumor cells were cultured for the next week for colony formation. The formed cellular colonies were subsequently quantified by crystal violet staining method.

### Wound healing experiment

Firstly, UBC tumor cells were transferred into plates (#354721, Corning, Inc., Corning, NY, USA) for 48 h, and scratch was then created by sterilized pipet tip. After purification with D-Hanks, samples were subsequently cultured in medium with 1% FBS. Images were respectively examined by digital camera at 0 and 48 hours after wound creation (triple randomly selected fields were imaged for each experimental group).

### Transwell assays

Cell invasion experiments were conducted utilizing Transwell chamber (Millipore, Burlington, MA, USA). Culture medium combined with 10% FBS was placed into the bottom chambers (#3268, Corning Inc., Corning, NY, USA), and each group of cells were firstly placed in culture medium and then placed into the top chamber. Then after 48 h of cells incubating at 37°C, cotton swab was used to remove those cells failed to migrate through the pores. Afterwards, Transwell chambers were fixed for 30 min using 4% paraformaldehyde, and were stained using 1% crystal violet (#C0775, Sigma-Aldrich, St. Louis, MO, USA) for 20 min. Finally, bottom chamber cells were quantified utilizing inverted phase-contrast microscope.

### Immunoprecipitation

For immunoprecipitation experiments, the abs were firstly treated with 30 μL of magnetic beads under rotation for 1.5 hours. Then GLB+ lysis buffer in combination of protease inhibitor agents were utilized for cell lysates extraction of each treatment group. Subsequently, cell lysates combined with antibody-conjugated beads were placed for 4°C overnight. Subsequently, the samples were cleaned 4 times using GLB+ lysis buffer. Afterwards, the sample proteins were resuspended and 6 × SDS-PAGE loading buffer was used for boiling 15 min. The boiled sample was put on ice for 2 min and was transferred to subsequent electrophoresis.

### Ubiquitination assay

Each experimental group of cells with 1 × 10^5^ density was firstly placed onto cell culture dishes. After 24 hours, 10 μM MG132 treatment was added with the cells for additional 12 hours. Cell samples were then cleaned twice by cold PBS and were then lysed using 10 ml of lysis buffer. Then 50 μl of Ni-NTA agarose beads (QIAGEN, Venlo, The Netherlands, Cat No. 30210) were added into cell samples, before sonication of the treated cell samples was performed. Cell samples were subsequently cultured for additional 4 hours at 4°C. Then, 15 ml of lysis buffer and wash buffer B were sequentially applied to purify samples. Afterwards, beads were eluted by adding 40 μl of elution buffer and samples were treated for another 15 min before centrifuge under 9000 rpm for 3 min. Finally, samples were boiled and transferred for following Western blot assay.

### Immunofluorescence

The experimental group of cells were firstly incubated using 4% paraformaldehyde and then treated with 5% BSA (#MB4219-3, meilunbio, Dalian, China) for blocking. The cell samples were then incubated with corresponding primary antibodies at 4°C overnight and incubated with secondary antibodies (ab150077, Abcam, Cambridge, UK) at 37°C for 1.5 hours. Subsequently, the cell samples were stained with DAPI for 15 min and examined by confocal microscope (Ultra-View Vox, Perkin-Elmer, Waltham, MA, USA). The relative staining intensity was quantified using Image J software.

### Western blotting

The proteins were initially lysed using radio-immunoprecipitation assay (RIPA) lysis buffer containing a protein phosphatase inhibitor (#MB12707, meilunbio, Dalian, China). The Bicinchoninic acid (BCA) method was employed to measure protein concentration, and an equal amount of protein was used in each group for 10% SDS-PAGE (sodium dodecyl sulfate–polyacrylamide gel electrophoresis). The proteins were then transferred to a PVDF (polyvinylidene difluoride) membrane. The membrane was blocked using TBST containing 5% non-fat milk for 2 hours. Subsequently, the membranes were incubated with primary antibodies overnight at 4°C, followed by secondary antibodies for 2 hours at room temperature. Finally, the target genes were detected using an enhanced chemiluminescence detection kit (Thermo Fisher, Waltham, MA, USA), and the bands were analyzed using ImageJ software. All antibodies used in this study are listed as follows. Anti-human UCHL1 Rabbit Monoclonal antibody (TA591048, TrueRAB, Inc., Rockville, MD, USA); Anti-human PKM2 Mouse Monoclonal Antibody (CF190266, TrueMAB, Inc., Rockville, MD, USA); Anti-human GAPDH Mouse Monoclonal Antibody (TA802519S, TrueMAB, Inc.); Anti-human Ki-67 Rat Monoclonal Antibody (TA801577, TrueMAB, Inc.); Anti-human PCNA Mouse Monoclonal Antibody (CF800894, TrueMAB, Inc.); Anti-human E-Cadherin Rabbit Monoclonal Antibody (TA592133, TrueRAB, Inc.); Anti-human N-Cadherin Mouse Monoclonal Antibody (TA503933, TrueMAB, Inc.); Anti-human Vimentin Rabbit Polyclonal antibody (TA383237, TrueRAB, Inc.); Anti-human mTOR Mouse Monoclonal Antibody (66888-1-Ig, Proteintech Group, Inc., Rosemont, IL, USA); Anti-human GLUT1 Rabbit Monoclonal Antibody (21829-1-AP, Proteintech Group, Inc.); Anti-human GLUT1 Mouse Monoclonal Antibody (CY5360, Abways, Shanghai, China); Anti-HA Mouse Monoclonal antibody (M180-3, MBL, Woburn, MA, USA); Anti-FLAG^®^ M2 Mouse Monoclonal antibody (F1804 Sigma-Aldrich); Normal Mouse IgG Polyclonal Antibody (12-371, Millipore); Anti-human 4EBP1 Mouse Monoclonal Antibody (60246-1-Ig, Proteintech Group, Inc.); Anti-human S6K Rabbit Monoclonal Antibody (14485-1-AP, Proteintech Group, Inc.); Anti-human Phospho-4E-BP1 (Thr70) Polyclonal Antibody (AY0690, Abways); Anti-human Phospho-S6K (Ser424) Polyclonal Antibody (CY6407, Abways).

### Statistical analysis

Statistical analysis was conducted utilizing GraphPad Prism 7. Student’s *t*-tests were applied to analyze statistical differences for numerical data comparison between two experimental groups, and one-way ANOVA method was applied to investigate significant differences among multiple groups. Differences were considered statistically significant at *p* < 0.05. Mean ± standard deviation was used to represent data.

## RESULTS

### UCHL1 upregulation is characteristic in UBC cancer tissue and correlated with inferior prognosis

**Figure 1:**
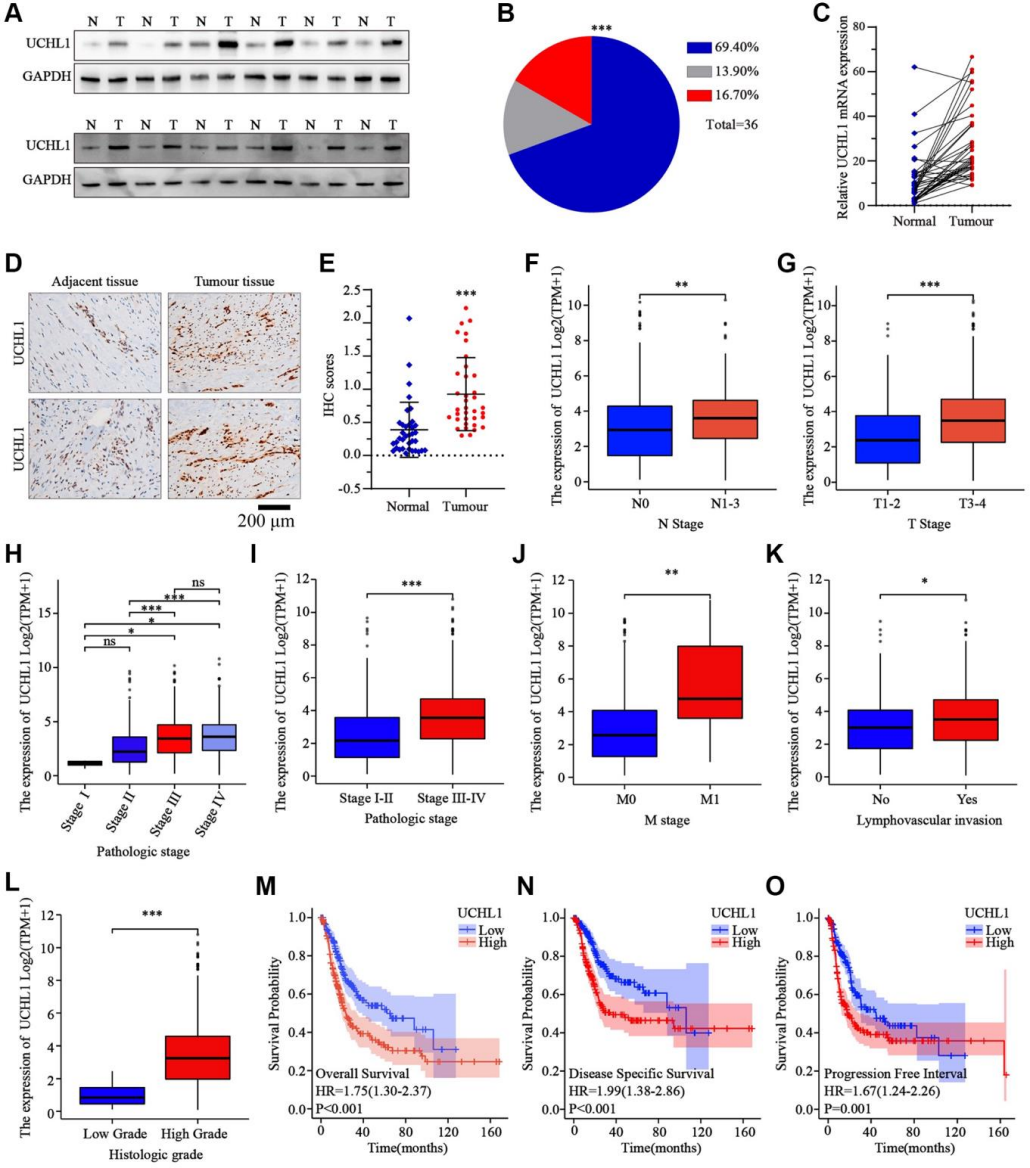
**The expression levels of UCHL1 are frequently up-regulated in UBC tissues and is positively associated with advanced clinical stage and poor outcomes in UBC patients.** (**A**, **B**) WB detection of UCHL1 protein expression pattern in 36 matched sample pairs of UBC tumor and adjacent normal tissues. (**C**) PCR detection of UCHL1 mRNA expression pattern in 36 matched sample pairs of UBC tumor and adjacent normal tissues. (**D**, **E**) IHC method detection and IHC score comparison of UCHL1 protein expression pattern in normal adjacent tissues and UBC tumor tissues (*n* = 3). (**F**, **G**) UCHL1 mRNA expression comparison between UBC patient subgroups with different N-T stages. (**H**–**L**) UCHL1 mRNA expression comparison between UBC patient subgroups with different pathological stages, M stages, lympho-vascular invasion status and histologic grade. (**M**–**O**) Kaplan-Meier analysis on the correlation of UCHL1 expression level with UBC patients’ prognostic endpoints including overall survival, disease-specific survival and progression free survival.

In this research, a total of 36 matched UBC tumor and adjacent normal tissue sample pairs were used for mRNA and protein level detection. As the results shown in Figure 1A–1C, the majority of UBC tumor tissues exhibited significantly increased expression of UCHL1 mRNA and protein level. Subsequent IHC assay also indicated consistent UCHL1 elevation in tumor tissues (Figure 1D, 1E). Next, in order to explore the impact of UCHL1 expression on UBC patients’ disease stage and prognosis, *in silico* analysis on public TCGA platform UBC tumor mRNA-seq datasets were subsequently performed. UBC tumor tissue mRNA sequencing and corresponding clinical stage and prognostic datasets were acquired. UBC patients were further divided into UCHL1-high and UCHL1-low subgroup based on UCHL1 level. As shown in Figure 1F–1L, we demonstrated that subgroup of patients with advanced N stages (N1-N3), M stage (M1), T stage (T3-T4) exhibited significantly elevated UCHL1 expression level. Additionally, patients with advanced pathologic stages, lymph-vascular invasion, and histologic stages exhibited significantly increased tumor UCHL1 expression level. As for the prognosis analysis, the results shown in Figure 1M–1O suggested that patients with high tumor UCHL1 expression level exhibited notably inferior prognostic factors, including overall survival, disease specific survival and progression free survival.

### UCHL1 modulation significantly impacts UBC tumor cell malignancy

**Figure 2:**
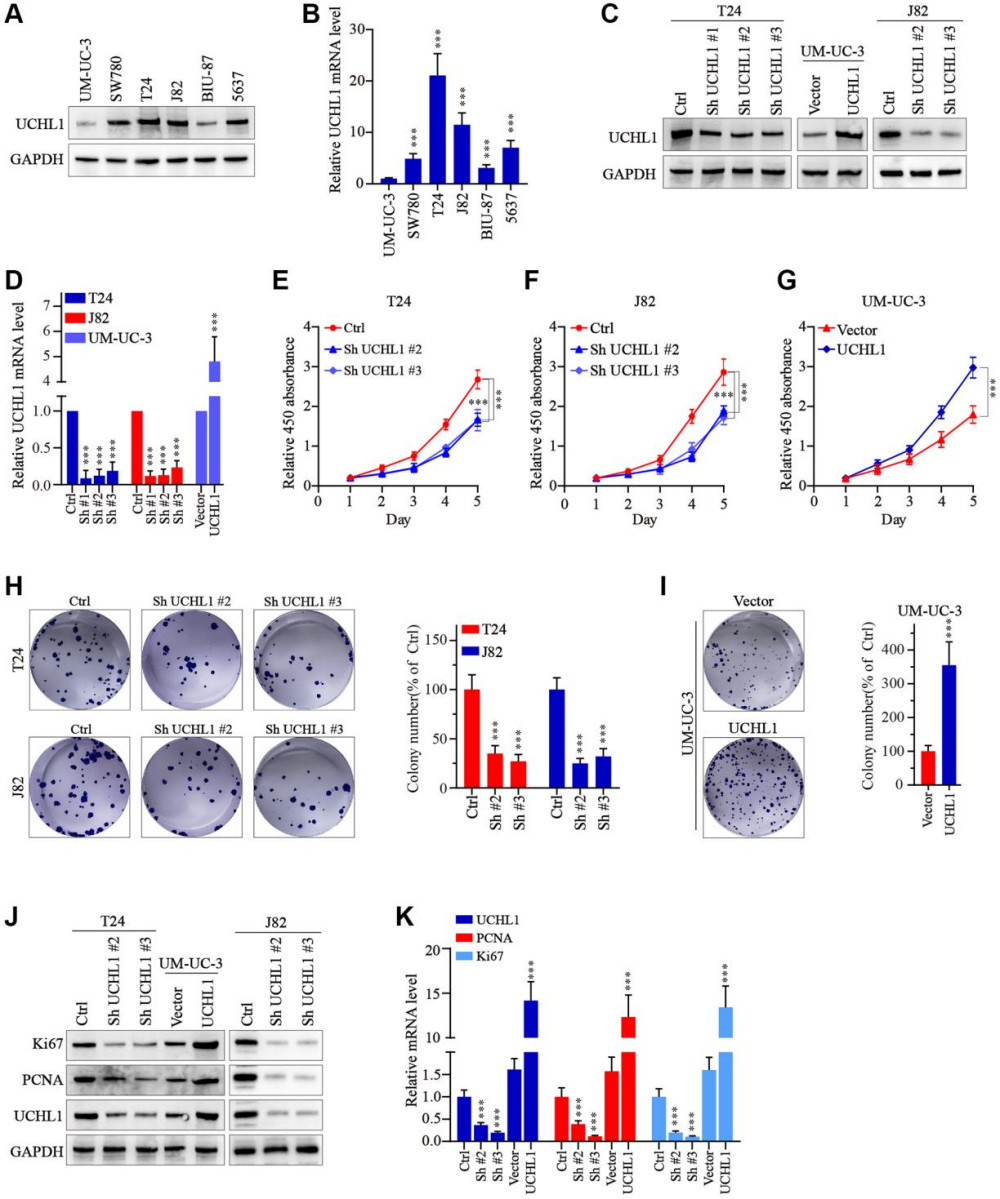
**UCHL1 promotes the proliferation capacity of UBC cells *in vitro*.** (**A**, **B**) WB and qRT-PCR tests on the UCHL1 protein and mRNA in several UBC cell lines including UM-UC-3, SW780, T24, J82, BIU-87 and 5637 (*n* = 3). (**C**, **D**) Design and WB/qRT-PRC functional validation assay of UCHL1-specific shRNAs and overexpression vectors (*n* = 3). (**E**–**G**) CCK8 proliferation assay to examine the impact of UCHL1 silencing or overexpression on the proliferative abilities of UBC cell lines including T24, J82 and UM-UC-3 (*n* = 3). (**H**, **I**) Colony formation assay to investigate the influences of UCHL1 silencing or overexpression on the proliferation of UBC cell lines (*n* = 3). (**J**, **K**) Ki67 and PCNA detection by WB and qRT-PCR method in UBC cell lines which were respectively treated by UCHL1-specific shRNAs or overexpression vectors (*n* = 3).

Then, to further explore the impact on the malignancy of UBC cancer cells, several UBC cell lines were utilized and UCHL1 mRNA and protein level were respectively examined. Results shown in Figure 2A, 2B suggested that UCHL1 was significantly upregulated in several UBC cell lines. To further modulate UCHL1 expression, UCHL1-specific shRNAs and overexpression vectors were designed and validated in T24/UM-UC-3/J82 cell line models (Figure 2C, 2D). Then cellular proliferation CCK8 in combination of colony formation assays were conducted and results indicated that UBC cells treated with UCHL1-specific shRNAs significantly suppressed tumor cell proliferation and colony formation. In contrast, UCHL1 overexpression significantly enhanced tumor cell expansion (Figure 2E–2I). Subsequent experiments also suggested that UCHL1 overexpression significantly enhanced Ki67 and PCNA protein and mRNA level, and *vice versa* (Figure 2J, 2K*).*

**Figure 3:**
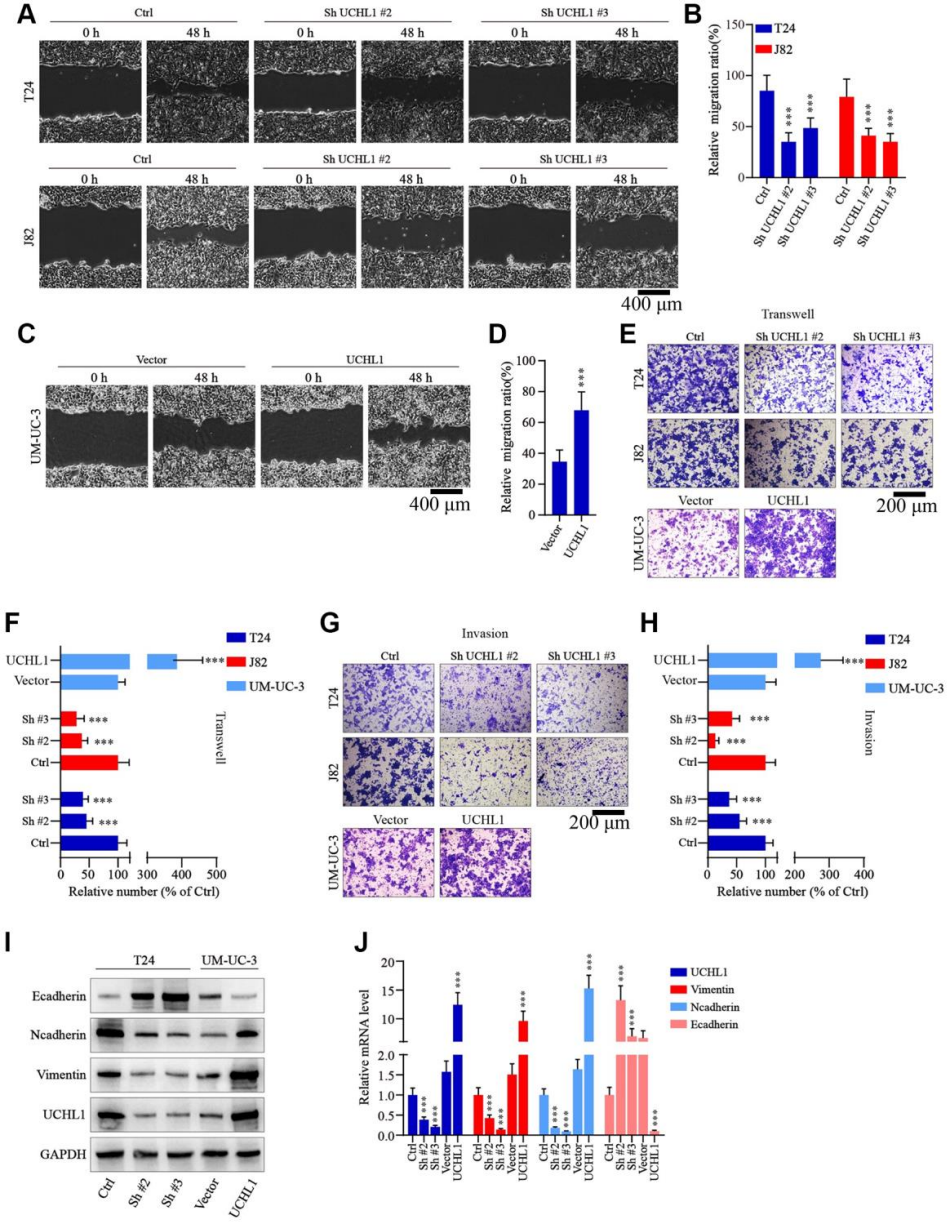
**UCHL1 promotes the motility capacity of UBC cells *in vitro*.** (**A**–**D**) Wound healing assay to investigate the influences of UCHL1 expression modulation on UBC tumor cell motility. Relative migration ratio of each cell group was statistically compared in bar charts (*n* = 3). (**E**–**H**) Transwell and cellular invasion assay to detect the impact of UCHL1 modulation on UBC tumor cell migration and invasion capabilities. Relative number of migrated or invaded cells of each group was statistically compared in bar charts (*n* = 3). (**I**, **J**) WB/qRT-PCR quantification of EMT biomarkers (E-cadherin, N-cadherin, Vimentin) in UBC cell groups transfected with UCHL1 shRNAs or overexpression vectors (*n* = 3).

Moreover, to examine the impact of UCHL1 modulation on tumor migration and invasion, wound healing assays in combination of Transwell/invasion assays were subsequently performed. As shown in Figure 3A–3D, UBC tumor cells with UCHL1 shRNAs treatment exhibited significantly suppressed relative migration ratio, compared with control group. In contrast, cell group with UCHL1 overexpression demonstrated significantly enhanced cellular migration ratio. Consistently, UCHL1 shRNAs treatment significantly impaired tumor cell migration and invasion in Transwell/cellular invasion experiments (Figure 3E–3H). Further molecular studies indicated that UCHL1 shRNAs treatment remarkably reversed tumor cellular EMT process, which promoted the expression of E-cadherin while reducing the expression level of N-cadherin and vimentin, thus UCHL1 silencing reducing the migrative capability of UBC tumor cells through inhibition of mesenchymal phenotypic transition (Figure 3I, 3J).

### UCHL1 regulates UBC tumor cell malignant phenotype through interaction with PKM2

**Figure 4:**
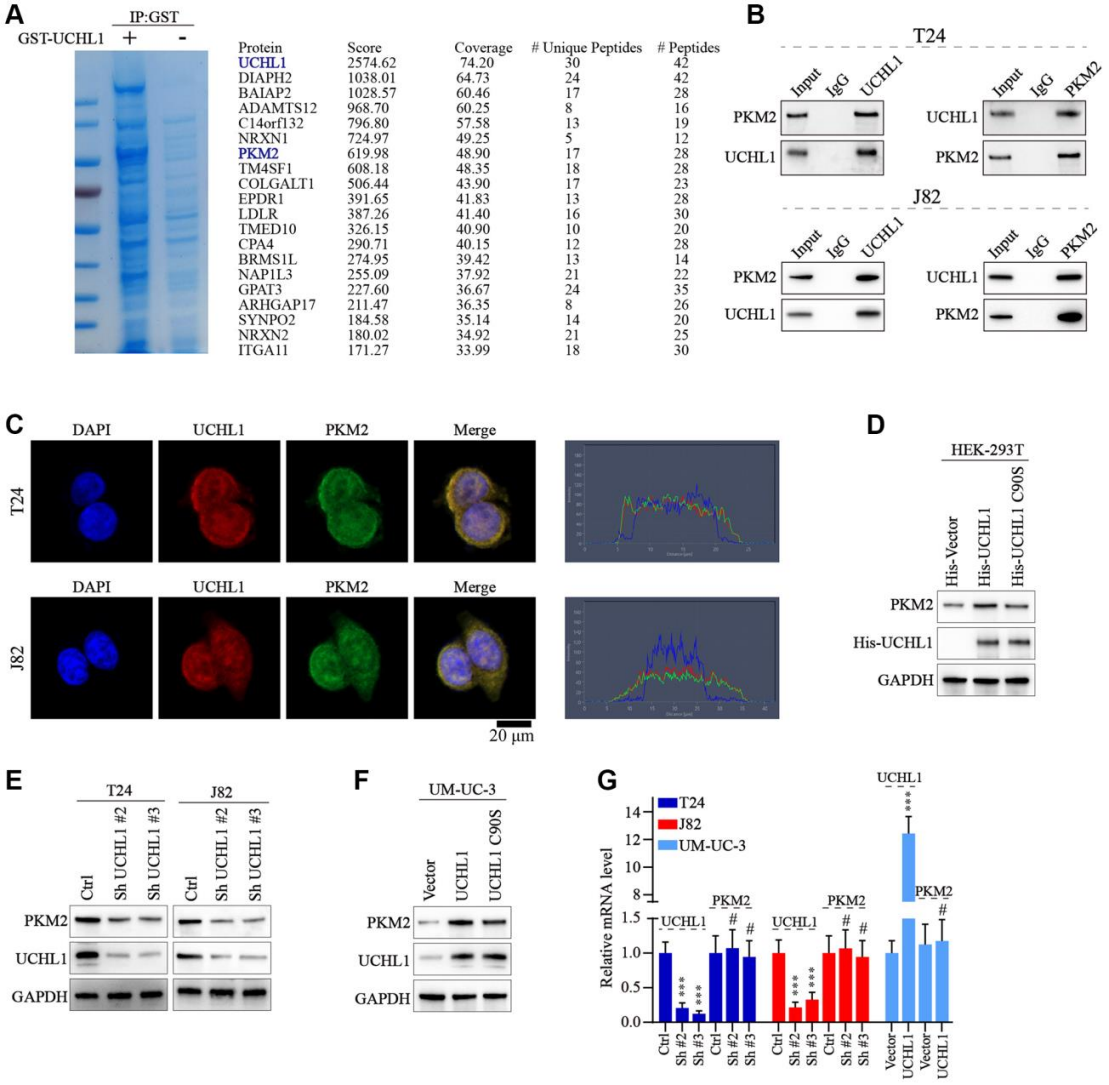
**UCHL1 interacts with PKM2 and upregulates the PKM2 protein level.** (**A**) Coomassie blue staining experiment on anti-UCHL1-immunoprecipitated cell protein samples, and Co-IP-MS assay was performed on samples immunoprecipitated by anti-UCHL1 antibody to investigate potential UCHL1 interacting protein targets. (**B**) Co-IP assay to confirm the protein-protein interaction of UCHL1 and PKM2 in T24 and J82 cell line models. (**C**) Immunofluorescence co-localization assay to confirm the protein interaction of UCHL1 and PKM2 in T24 and J82 cell line models. (**D**) Evaluation of UCHL1 regulatory role on PKM2 by exogenously transfection of His-tagged UCHL1 and His-tagged loss-of-function UCHL1 C90S mutated vector into HEK-293T cells. (**E**, **F**) Explore the impact of UCHL1 silencing or overexpression on PKM2 expression in UBC cancer cell line models by transfection of UCHL1-specific shRNAs or overexpression vectors. (**G**) PCR detection of PKM2 mRNA level modulated by UCHL1 in different UBC cell line groups respectively transfected with UCHL1-specific shRNAs or overexpression vectors (*n* = 3).

Next, we intended to further discover the underlying mechanism of UCHL1 regulatory function. GST pull-down assay followed by IP-MS technique was used to detect potential UCHL1 interacting protein targets in UBC cell line lysate samples. As shown in Figure 4A, 4B, multiple target proteins were identified with potential UCHL1-interacting capabilities. Our attention focused on PKM2 as its regulatory role in multiple malignant diseases was identified in previous research [18]. Then Co-IP method was further applied and results indicated PKM2 protein can be detected in UCHL1-immunoprecipitated T24/J82 cell lysate samples, and *vice versa.* In addition, immunofluorescent colocalization assay further provided evidences for UCHL1-PKM2 interaction (Figure 4C). To understand the protein interaction detail of UCHL1-PKM2, we designed His-tagged vector of UCHL1 wildtype and UCHL1 C90S mutated vectors and transfected them into HEK-293T cell model respectively. As a result, for HEK-293T cell group transfected with wildtype His-UCHL1, significantly increased PKM2 level was detected, while for cell group transfected with mutated UCHL1 vector, no significant alteration of PKM2 protein level was identified (Figure 4D). Furthermore, wildtype/mutated UCHL1 overexpression vectors, in combination of UCHL1 specific shRNAs were subsequently transfected into UBC cell line groups. And results shown in Figure 4E, 4F indicated that UCHL1 shRNAs transfection significantly suppressed PKM2 level, while wildtype UCHL1 overexpression vector transfection promoted PKM2 expression. And such effects were diminished when mutated UCHL1 vectors were transfected. Interestingly, when detecting the mRNA level change of PKM2 regulated by UCHL1, we found that UCHL1 shRNAs and overexpression vector transfection significantly modulated UCHL1 mRNAs, while had no significant impact on PKM2 mRNA expression level (Figure 4G). The above results indicated that potential post-transcriptional regulatory function on PKM2 protein expression existed for UCHL1.

### UCHL1 promotes UBC cellular malignancy by inhibiting PKM2 protein degradation via ubiquitination pathway

**Figure 5:**
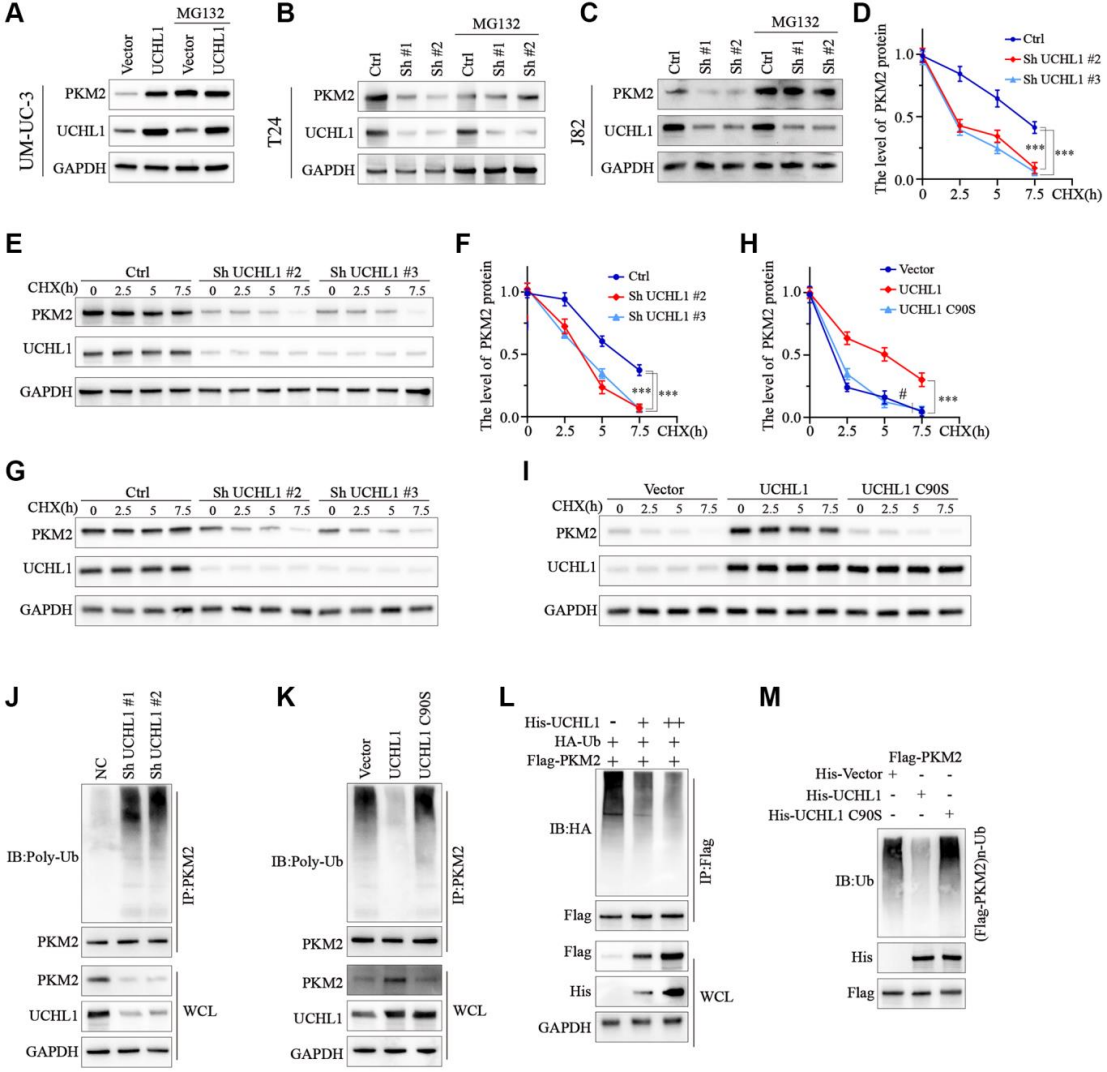
**UCHL1 upregulates the expression of PKM2 in UBC cells via de-ubiquitination modulation.** (**A**–**C**) WB assay on protein samples from UBC cell line groups respectively transfected with UCHL1 overexpression vector or shRNAs, in combination with or without MG132, in order to explore the impact of UCHL1 modulation on PKM2. (**D**–**I**) CHX chase experiment to detect PKM2 protein level degradation in UBC cell lines treated with UCHL1 shRNAs or UCHL1 wildtype/mutated overexpression vectors (*n* = 3). (**J**, **K**) PKM2 ubiquitination level detection using CO-IP method in UBC cell line groups treated with UCHL1 shRNAs or UCHL1 wildtype/mutated overexpression vectors. (**L**) PKM2 ubiquitination level detection using CO-IP method in HEK-293T cell line groups exogenously transfected with escalating dosage of His-tagged UCHL1 vectors, in combination of Flag-tagged PKM2 vectors and HA-tagged ubiquitin. (**M**) PKM2 ubiquitination level detection using CO-IP method in HEK-293T cell line groups exogenously transfected with His-tagged wildtype or mutated UCHL1 vectors, or control vectors, in combination of Flag-tagged PKM2 vectors.

To test the role of ubiquitination process in the regulation of PKM2 protein. UBC cell line groups transfected with UCHL1 overexpression or shRNAs were treated with or without MG132. As shown in Figure 5A, for UM-UC-3 cell group transfected with UCHL1 overexpression vectors, PKM2 protein level was significantly raised, while MG132 treatment further promoted PKM2 protein level. In contrast, for J82 and T24 cell groups treated with UCHL1 shRNAs, addition of MG132 treatment reversed the PKM2 suppression effects caused by UCHL1 silencing (Figure 5B, 5C). Further CHX chase experiment also indicated that UCHL1 silencing significantly enhanced the PKM2 protein degradation velocity in T24 and J82 cell line models (Figure 5D–5G). Meanwhile, wildtype UCHL1 overexpression vector transfection remarkably delayed the PKM2 protein degradation and raised its expression level, and mutated vectors didn’t exert same impact on PKM2 proteins (Figure 5H, 5I). Subsequently, immunoprecipitation method was further used to detect PKM2 ubiquitination level regulated by UCHL1, and the results indicated that UCHL1 silencing by shRNAs transfection significantly elevated the ubiquitination of PKM2 protein, detected by WB method in PKM2 immunoprecipitated samples (Figure 5J). In comparison, wildtype UCHL1 overexpression vector treatment reduced PKM2 ubiquitination, and mutated UCHL1 vector transfection exerted no significant influences on PKM2 poly-ubiquitin levels (Figure 5K). Additionally, HEK-293T cell model was also used for exogenously transfection of escalating dosage of His-tagged UCHL1 wildtype vectors, in combination of HA-tagged ubiquitin and Flag-tagged PKM2 vectors. Results indicated that HA-Ubiquitin level was decreased dose-dependently in cell groups transfected with His-UCHL1 vectors (Figure 5L). In contrast, when mutated His-tagged UCHL1 vectors were transfected in combination of Flag-tagged PKM2 vectors, no significant change of Flag-PKM2-Ub level was detected (Figure 5M).

**Figure 6:**
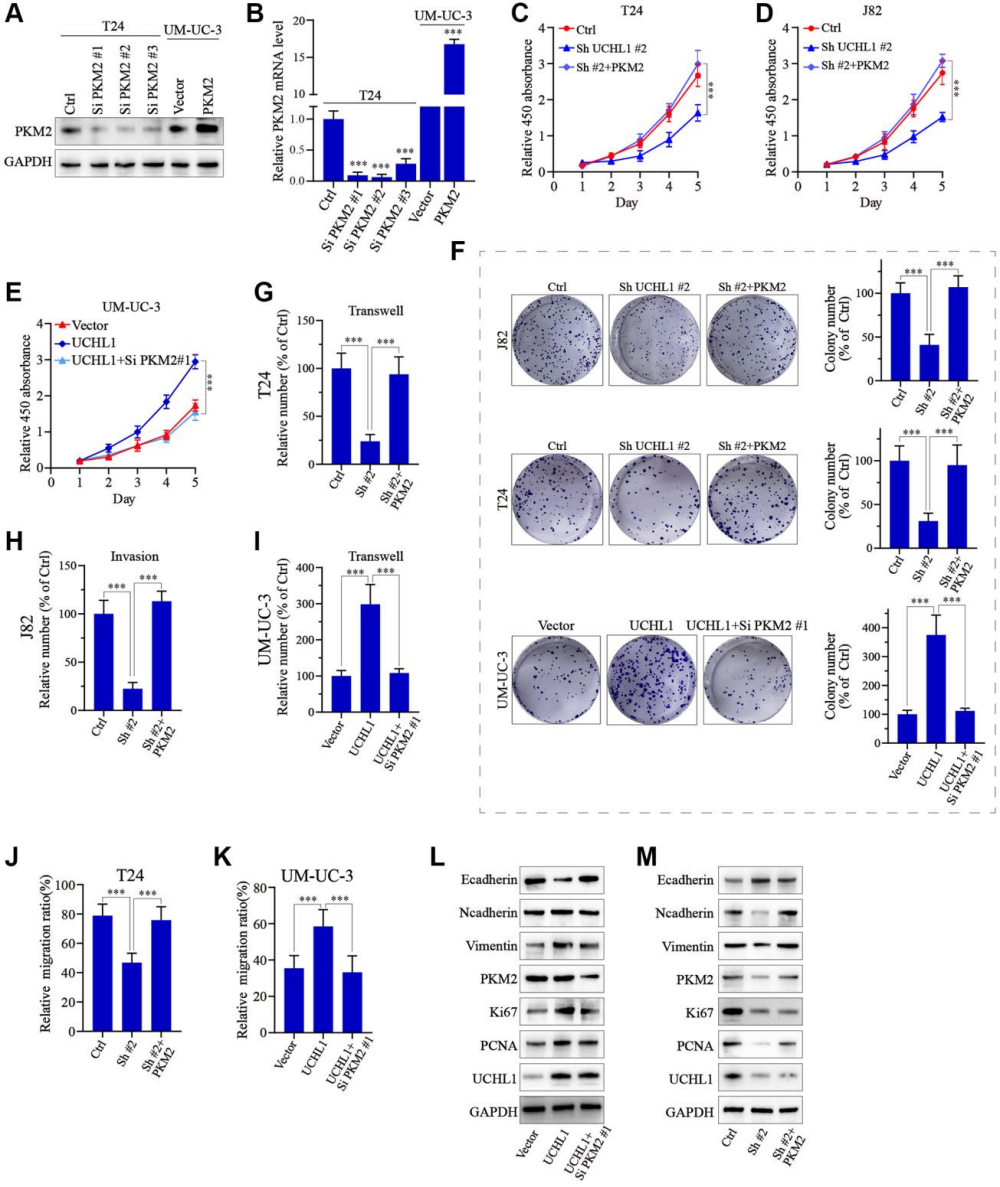
**UCHL1 affects proliferation and migration capacity of UBC cells through PKM2 regulation.** (**A**, **B**) Design and functional validation of PKM2 specific siRNA and overexpression vectors in UBC cancer cell line (T24 and UM-UC-3) via WB/PCR method. (**C**–**E**) CCK8 assay on UBC cell line groups respectively transfected with UCHL1 shRNA/overexpression vector in combination of PKM2 overexpression/siRNAs (*n* = 3). (**F**) Colony formation assay on UBC cell line groups respectively transfected with UCHL1 shRNAs/overexpression vector alone or in combination of PKM2 siRNA/overexpression vectors (*n* = 3). (**G**–**I**) Transwell and cellular invasion assay on UBC cell line groups transfected with UCHL1 shRNAs/overexpression alone or in combination with PKM2 siRNAs/overexpression vectors (*n* = 3). (**J**, **K**) Wound healing experiments on UBC cell line groups transfected with UCHL1 shRNAs/overexpression vector alone or in combination of PKM2 overexpression/siRNAs (*n* = 3). (**L**, **M**) WB investigation on EMT biomarkers and proliferation biomarkers in UBC cell line groups transfected UCHL1 shRNAs/overexpression vector alone or in combination of PKM2 overexpression/siRNAs.

To further confirm our findings, PKM2 specific siRNAs and overexpression vectors were utilized and tested their modulative effects on PKM2 expression in UBC tumor cell line models (Figure 6A, 6B). Then PKM2 overexpression vectors/siRNAs were transfected into T24 and J82 cells with or without UCHL1 shRNAs to explore PKM2 regulation on UBC tumor cellular proliferation. As shown in Figure 6C–6F, CCK8 assay and colony formation assay results showed that UCHL1 shRNAs treatment remarkably reduced tumor cell proliferation, while concomitant transfection of PKM2 overexpression vectors reversed the effects of UCHL1 shRNAs, and *vice versa.* Subsequently, we detected the impact of PKM2 modulation on cellular mobility using Transwell and wound healing assay. Transwell results shown in Figure 6G–6I and Figure 6A–6C combined with wound healing assay results shown in Figure 6J, 6K and Figure 6D, 6E both suggested that UCHL1 silencing significantly reduced tumor cell migration and invasion capabilities, while concomitant treatment with PKM2 overexpression vector completely reversed such effects. Similarly, combinatory PKM2 siRNAs treatment also reversed the promotive influences on tumor migration and invasion provided by UCHL1 overexpression vector transfection alone. Following molecular study on EMT and cellular proliferation gene biomarkers also suggested that PKM2 siRNA concomitant treatment can totally abrogate the impact of UCHL1 overexpression vector on UBC tumor cells towards more proliferative, mesenchymal phenotype, which is characterized by reduced E-cadherin and increased N-cadherin, Vimentin, Ki67 and PCNA (Figure 6L, 6M).

## DISCUSSION

In this study, we reported for the first time that UCHL1 upregulation is characteristic in UBC cancer cells and it plays an oncogenic role in the tumor progression and invasion. Consistently, previous researches have indicated that UCHL1 has promotive effects in carcinogenesis. For example, high expression of UCHL1 was noted in Burkitt’s lymphoma and chronic lymphocytic lymphoma cell lines [19, 20]. And aberrant UCHL1 expression enhances lymphomagenesis through AKT pathway, by inhibition antagonistic enzyme PHLPP1 mediated by UCHL1’s DUB activity [21]. In addition, another study on lung cancer suggested that UCHL1 expression is significantly upregulated in non-small-cell-lung cancer cells and it notably enhanced tumor invasion and metastasis in both *in vitro* and *in vivo* models through activation of AKT pathway [22]. However, in contrast, other researchers have reported the opposite observations. For instance, in nasopharyngeal carcinoma, UCHL1 enhances p53 signaling pathway and serves as a tumor suppressor which is often silenced during carcinogenesis [23]. Besides, UCHL1 has also been shown to have tumor suppressive effects in prostate cancer and breast cancer in other studies [24, 25]. Therefore, based on the above findings, the regulatory role of UCHL1 in cancer pathogenesis and progression is highly complex and tumor-type dependent, and more detailed studies will be needed to further decipher the controversial role of UCHL1.

Furthermore, our study demonstrated for the first time that UCHL1 interacted with PKM2 to exert its oncogenic activity. Pyruvate kinase M2 (PKM2), is a member of PKM gene family which encodes pyruvate kinase (PK) and functions as a rate-limiting enzyme for cellular glycolysis [26]. In comparison of constituently high-level expression of PKM1, the expression of PKM2 is complexly regulated, in order to adapt to various cellular physiological states [27]. Current researches have confirmed that dimeric PKM2 assists tumor cells to get energy under hypoxic conditions [28], which enhances tumor proliferation. Besides, PKM2 in nucleus can reduce CDH1 gene encoding E-cadherin, and facilitating EMT and tumor cell invasion [29, 30]. The results of our study also confirmed the above evidences and further expanded the regulatory network of PKM2. Furthermore, other studies indicated that dimeric PKM2 also regulates glycolysis process of tumor associated macrophages (TAMs) in tumor microenvironment (TME), and mediates the transition of TAM1 to TAM2 subtype, which promotes various tumor metastasis and invasion [31, 32]. These results broaden the modulative roles of PKM2 in both tumor cellular metabolism and anti-tumor immunity regulation.

## CONCLUSIONS

In summary, our study for the first time demonstrated UCHL1 high-expression as a characteristic in UBC tumor cells, and UCHL1-PKM2 interaction played an interesting role in UBC tumor cell proliferation, migration and metastasis. Our study suggested PKM2-targeted treatment might have a potential value in metastatic malignancy therapy development in the future.

## References

[1] Antoni S, Ferlay J, Soerjomataram I, Znaor A, Jemal A, Bray F. Bladder Cancer Incidence and Mortality: A Global Overview and Recent Trends. Eur Urol. 2017; 71:96–108. 10.1016/j.eururo.2016.06.010 27370177

[2] Willis D, Kamat AM. Nonurothelial bladder cancer and rare variant histologies. Hematol Oncol Clin North Am. 2015; 29:237–52. 10.1016/j.hoc.2014.10.011 25836932

[3] Kamat AM, Hahn NM, Efstathiou JA, Lerner SP, Malmström PU, Choi W, Guo CC, Lotan Y, Kassouf W. Bladder cancer. Lancet. 2016; 388:2796–810. 10.1016/S0140-6736(16)30512-8 27345655

[4] Jani AB, Efstathiou JA, Shipley WU. Bladder preservation strategies. Hematol Oncol Clin North Am. 2015; 29:289–300. 10.1016/j.hoc.2014.10.004 25836935

[5] Witjes JA, Compérat E, Cowan NC, De Santis M, Gakis G, Lebret T, Ribal MJ, Van der Heijden AG, Sherif A, and European Association of Urology. EAU guidelines on muscle-invasive and metastatic bladder cancer: summary of the 2013 guidelines. Eur Urol. 2014; 65:778–92. 10.1016/j.eururo.2013.11.046 24373477

[6] Milowsky MI, Rumble RB, Booth CM, Gilligan T, Eapen LJ, Hauke RJ, Boumansour P, Lee CT. Guideline on Muscle-Invasive and Metastatic Bladder Cancer (European Association of Urology Guideline): American Society of Clinical Oncology Clinical Practice Guideline Endorsement. J Clin Oncol. 2016; 34:1945–52. 10.1200/JCO.2015.65.9797 27001593

[7] Clark PE, Spiess PE, Agarwal N, Bangs R, Boorjian SA, Buyyounouski MK, Efstathiou JA, Flaig TW, Friedlander T, Greenberg RE, Guru KA, Hahn N, Herr HW, et al. NCCN Guidelines Insights: Bladder Cancer, Version 2.2016. J Natl Compr Canc Netw. 2016; 14:1213–24. 10.6004/jnccn.2016.0131 27697976 PMC5379654

[8] Chang SS, Boorjian SA, Chou R, Clark PE, Daneshmand S, Konety BR, Pruthi R, Quale DZ, Ritch CR, Seigne JD, Skinner EC, Smith ND, McKiernan JM. Diagnosis and Treatment of Non-Muscle Invasive Bladder Cancer: AUA/SUO Guideline. J Urol. 2016; 196:1021–9. 10.1016/j.juro.2016.06.049 27317986

[9] Babjuk M, Böhle A, Burger M, Capoun O, Cohen D, Compérat EM, Hernández V, Kaasinen E, Palou J, Rouprêt M, van Rhijn BWG, Shariat SF, Soukup V, et al. EAU Guidelines on Non-Muscle-invasive Urothelial Carcinoma of the Bladder: Update 2016. Eur Urol. 2017; 71:447–61. 10.1016/j.eururo.2016.05.041 27324428

[10] Sternberg CN, Skoneczna I, Kerst JM, Albers P, Fossa SD, Agerbaek M, Dumez H, de Santis M, Théodore C, Leahy MG, Chester JD, Verbaeys A, Daugaard G, et al, and European Organisation for Research and Treatment of Cancer Genito-Urinary Cancers Group, and Groupe d'Etude des Tumeurs Urogénitales, and National Cancer Research Institute Bladder Cancer Study Group, and National Cancer Institute of Canada Clinical Trials Group, and German Association of Urologic Oncology. Immediate versus deferred chemotherapy after radical cystectomy in patients with pT3-pT4 or N+ M0 urothelial carcinoma of the bladder (EORTC 30994): an intergroup, open-label, randomised phase 3 trial. Lancet Oncol. 2015; 16:76–86. 10.1016/S1470-2045(14)71160-X 25498218

[11] Advanced Bladder Cancer Meta-analysis Collaboration. Neoadjuvant chemotherapy in invasive bladder cancer: a systematic review and meta-analysis. Lancet. 2003; 361:1927–34. 10.1016/s0140-6736(03)13580-5 12801735

[12] Winquist E, Kirchner TS, Segal R, Chin J, Lukka H, and Genitourinary Cancer Disease Site Group, Cancer Care Ontario Program in Evidence-based Care Practice Guidelines Initiative. Neoadjuvant chemotherapy for transitional cell carcinoma of the bladder: a systematic review and meta-analysis. J Urol. 2004; 171:561–9. 10.1097/01.ju.0000090967.08622.33 14713760

[13] Van Allen EM, Mouw KW, Kim P, Iyer G, Wagle N, Al-Ahmadie H, Zhu C, Ostrovnaya I, Kryukov GV, O'Connor KW, Sfakianos J, Garcia-Grossman I, Kim J, et al. Somatic ERCC2 mutations correlate with cisplatin sensitivity in muscle-invasive urothelial carcinoma. Cancer Discov. 2014; 4:1140–53. 10.1158/2159-8290.CD-14-0623 25096233 PMC4238969

[14] Plimack ER, Dunbrack RL, Brennan TA, Andrake MD, Zhou Y, Serebriiskii IG, Slifker M, Alpaugh K, Dulaimi E, Palma N, Hoffman-Censits J, Bilusic M, Wong YN, et al. Defects in DNA Repair Genes Predict Response to Neoadjuvant Cisplatin-based Chemotherapy in Muscle-invasive Bladder Cancer. Eur Urol. 2015; 68:959–67. 10.1016/j.eururo.2015.07.009 26238431 PMC4764095

[15] Dadhania V, Zhang M, Zhang L, Bondaruk J, Majewski T, Siefker-Radtke A, Guo CC, Dinney C, Cogdell DE, Zhang S, Lee S, Lee JG, Weinstein JN, et al. Meta-Analysis of the Luminal and Basal Subtypes of Bladder Cancer and the Identification of Signature Immunohistochemical Markers for Clinical Use. EBioMedicine. 2016; 12:105–17. 10.1016/j.ebiom.2016.08.036 27612592 PMC5078592

[16] Choi W, Porten S, Kim S, Willis D, Plimack ER, Hoffman-Censits J, Roth B, Cheng T, Tran M, Lee IL, Melquist J, Bondaruk J, Majewski T, et al. Identification of distinct basal and luminal subtypes of muscle-invasive bladder cancer with different sensitivities to frontline chemotherapy. Cancer Cell. 2014; 25:152–65. 10.1016/j.ccr.2014.01.009 24525232 PMC4011497

[17] Mondal M, Conole D, Nautiyal J, Tate EW. UCHL1 as a novel target in breast cancer: emerging insights from cell and chemical biology. Br J Cancer. 2022; 126:24–33. 10.1038/s41416-021-01516-5 34497382 PMC8727673

[18] Zhu S, Guo Y, Zhang X, Liu H, Yin M, Chen X, Peng C. Pyruvate kinase M2 (PKM2) in cancer and cancer therapeutics. Cancer Lett. 2021; 503:240–8. 10.1016/j.canlet.2020.11.018 33246091

[19] Gavioli R, Frisan T, Vertuani S, Bornkamm GW, Masucci MG. c-myc overexpression activates alternative pathways for intracellular proteolysis in lymphoma cells. Nat Cell Biol. 2001; 3:283–8. 10.1038/35060076 11231578

[20] Ovaa H, Kessler BM, Rolén U, Galardy PJ, Ploegh HL, Masucci MG. Activity-based ubiquitin-specific protease (USP) profiling of virus-infected and malignant human cells. Proc Natl Acad Sci U S A. 2004; 101:2253–8. 10.1073/pnas.0308411100 14982996 PMC356937

[21] Hussain S, Foreman O, Perkins SL, Witzig TE, Miles RR, van Deursen J, Galardy PJ. The de-ubiquitinase UCH-L1 is an oncogene that drives the development of lymphoma in vivo by deregulating PHLPP1 and Akt signaling. Leukemia. 2010; 24:1641–55. 10.1038/leu.2010.138 20574456 PMC3236611

[22] Kim HJ, Kim YM, Lim S, Nam YK, Jeong J, Kim HJ, Lee KJ. Ubiquitin C-terminal hydrolase-L1 is a key regulator of tumor cell invasion and metastasis. Oncogene. 2009; 28:117–27. 10.1038/onc.2008.364 18820707

[23] Li L, Tao Q, Jin H, van Hasselt A, Poon FF, Wang X, Zeng MS, Jia WH, Zeng YX, Chan AT, Cao Y. The tumor suppressor UCHL1 forms a complex with p53/MDM2/ARF to promote p53 signaling and is frequently silenced in nasopharyngeal carcinoma. Clin Cancer Res. 2010; 16:2949–58. 10.1158/1078-0432.CCR-09-3178 20395212

[24] Xiang T, Li L, Yin X, Yuan C, Tan C, Su X, Xiong L, Putti TC, Oberst M, Kelly K, Ren G, Tao Q. The ubiquitin peptidase UCHL1 induces G0/G1 cell cycle arrest and apoptosis through stabilizing p53 and is frequently silenced in breast cancer. PLoS One. 2012; 7:e29783. 10.1371/journal.pone.0029783 22279545 PMC3261155

[25] Ummanni R, Jost E, Braig M, Lohmann F, Mundt F, Barett C, Schlomm T, Sauter G, Senff T, Bokemeyer C, Sültmann H, Meyer-Schwesinger C, Brümmendorf TH, Balabanov S. Ubiquitin carboxyl-terminal hydrolase 1 (UCHL1) is a potential tumour suppressor in prostate cancer and is frequently silenced by promoter methylation. Mol Cancer. 2011; 10:129. 10.1186/1476-4598-10-129 21999842 PMC3212821

[26] Witney TH, James ML, Shen B, Chang E, Pohling C, Arksey N, Hoehne A, Shuhendler A, Park JH, Bodapati D, Weber J, Gowrishankar G, Rao J, et al. PET imaging of tumor glycolysis downstream of hexokinase through noninvasive measurement of pyruvate kinase M2. Sci Transl Med. 2015; 7:310ra169. 10.1126/scitranslmed.aac6117 26491079

[27] Gui DY, Lewis CA, Vander Heiden MG. Allosteric regulation of PKM2 allows cellular adaptation to different physiological states. Sci Signal. 2013; 6:pe7. 10.1126/scisignal.2003925 23423437

[28] Chinopoulos C. OXPHOS Defects Due to mtDNA Mutations: Glutamine to the Rescue! Cell Metab. 2018; 27:1165–7. 10.1016/j.cmet.2018.05.010 29874564

[29] Amin S, Yang P, Li Z. Pyruvate kinase M2: A multifarious enzyme in non-canonical localization to promote cancer progression. Biochim Biophys Acta Rev Cancer. 2019; 1871:331–41. 10.1016/j.bbcan.2019.02.003 30826427

[30] Hamabe A, Konno M, Tanuma N, Shima H, Tsunekuni K, Kawamoto K, Nishida N, Koseki J, Mimori K, Gotoh N, Yamamoto H, Doki Y, Mori M, Ishii H. Role of pyruvate kinase M2 in transcriptional regulation leading to epithelial-mesenchymal transition. Proc Natl Acad Sci U S A. 2014; 111:15526–31. 10.1073/pnas.1407717111 25313085 PMC4217454

[31] Li X, Deng S, Liu M, Jin Y, Zhu S, Deng S, Chen J, He C, Qin Q, Wang C, Zhao G. The responsively decreased PKM2 facilitates the survival of pancreatic cancer cells in hypoglucose. Cell Death Dis. 2018; 9:133. 10.1038/s41419-017-0158-5 29374159 PMC5833844

[32] Hou PP, Luo LJ, Chen HZ, Chen QT, Bian XL, Wu SF, Zhou JX, Zhao WX, Liu JM, Wang XM, Zhang ZY, Yao LM, Chen Q, et al. Ectosomal PKM2 Promotes HCC by Inducing Macrophage Differentiation and Remodeling the Tumor Microenvironment. Mol Cell. 2020; 78:1192–206.e10. 10.1016/j.molcel.2020.05.004 32470318

